# Camera-based heart rate estimation for hospitalized newborns in the presence of motion artifacts

**DOI:** 10.1186/s12938-021-00958-5

**Published:** 2021-12-04

**Authors:** Qiong Chen, Yalin Wang, Xiangyu Liu, Xi Long, Bin Yin, Chen Chen, Wei Chen

**Affiliations:** 1 Center for Intelligent Medical Electronics, School of Information Science and Technology, Fudan University, Shanghai, China; 2 School of Art Design and Media, East China University of Science and Technology, Shanghai, China; 3 Department of Electrical Engineering, Eindhoven University of Technology, Eindhoven, The Netherlands; 4 Connected Care and Personal Health Department, Philips Research, Shanghai, China; 5 Human Phenome Institute, Fudan University, Shanghai, China

**Keywords:** Biomedical signal processing, Heart rate, Motion artifacts, Eulerian video magnification, Remote photoplethysmography (rPPG)

## Abstract

**Background:**

Heart rate (HR) is an important *vital sign* for evaluating the physiological condition of a newborn infant. Recently, for measuring HR, novel RGB camera-based non-contact techniques have demonstrated their specific superiority compared with other techniques, such as dopplers and thermal cameras. However, they still suffered poor robustness in infants’ HR measurements due to frequent body movement.

**Methods:**

This paper introduces a framework to improve the robustness of infants’ HR measurements by solving motion artifact problems. Our solution is based on the following steps: morphology-based filtering, region-of-interest (ROI) dividing, Eulerian video magnification and majority voting. In particular, ROI dividing improves ROI information utilization. The majority voting scheme improves the statistical robustness by choosing the HR with the highest probability. Additionally, we determined the dividing parameter that leads to the most accurate HR measurements. In order to examine the performance of the proposed method, we collected 4 hours of videos and recorded the corresponding electrocardiogram (ECG) of 9 hospitalized neonates under two different conditions—*rest still* and *visible movements*.

**Results:**

Experimental results indicate a promising performance: the mean absolute error during *rest still* and *visible movements* are 3.39 beats per minute (BPM) and 4.34 BPM, respectively, which improves at least 2.00 and 1.88 BPM compared with previous works. The Bland-Altman plots also show the remarkable consistency of our results and the HR derived from the ground-truth ECG.

**Conclusions:**

To the best of our knowledge, this is the first study aimed at improving the robustness of neonatal HR measurement under motion artifacts using an RGB camera. The preliminary results have shown the promising prospects of the proposed method, which hopefully reduce neonatal mortality in hospitals.

## Background

Newborn infants are prone to bradycardia [1], which induced by a variety of reasons, such as congenital heart disease [2] and electrolyte disorders [3]. The uncommon disorders may cause life-threatening problems that are difficult to diagnose early due to the different characteristics and clinical manifestations between neonates and older children. Therefore, as an essential physiological indicator, heart rate (HR) is vital for monitoring the health of newborns.

Contact HR measurement methods, such as electrocardiography measured by electrocardiogram (ECG) electrodes [4] and photoplethysmography (PPG) measured by pulse oximeters [5], have inherent limitations. First, repetitive removal and attachment of the electrodes make HR measurements cumbersome and inconvenient when clinical activities, such as physical examinations, are being performed [6]. Second, the skin of newborn babies are fragile and sensitive. Adhesive electrodes or gel may cause skin irritation and damage, which is adverse to the health and development of babies [7]. Third, the conductive gel has the possibility to solidify, which may affect the signal quality. In recent years, non-contact HR measurement techniques (including dopplers [8, 9], white noise [10], thermal/infrared cameras [11, 12] and RGB cameras [13, 14]) have proven effective in solving the problem of contact HR monitoring methods because of their unobtrusiveness and lack of skin contact. Among non-contact equipment, RGB cameras are the most popular due to their low-cost and high resolution. Dopplers and infrared cameras are more expensive compared with commercial RGB cameras, whereas the white noise solution is unsuitable for long-term (e.g., 24 h) monitoring due to the annoying sounds it produces.

The principle of RGB camera-based HR measurements [15] (which is also known as remote PPG) is based on the absorption of specific wavelengths of light by oxyhemoglobin and hemoglobin in blood vessels, while the surrounding tissues cannot do. During each heartbeat, changes in blood volume cause regulated light transmission and reflection, contributing to subtle skin color changes that are invisible to the naked eye but can be captured by an RGB camera. In practical scenarios, the face [16, 17] is usually spotted by the RGB camera as the region-of-interest (ROI). One reason for this is that the face skin is relatively thin and close to blood vessels, thus possessing positive measuring performance. The other reason is that the face is most visible compared with other parts of the body (e.g., arms or legs, which are often covered by a blanket). However, motion artifacts [11, 18, 19] are one of the main challenges influencing the robustness of HR estimation. For neonates, this is even more challenging as they move frequently and their movements are difficult to predict and control. In this work, limited motion types, that is, head rotation and non-rigid motions (e.g., eye blinking and emotion expressing) are considered due to babies’ lack of mobility. Recently, some proposed techniques have attempted to overcome the problem of motion artifacts on adults using RGB camera [18, 20, 21]. For example, Yu et al. [18] tackled the motion artifact problem during exercise by presenting a new artifact-reduction method consisting of planar motion compensation and blind source separation. Li et al. [20] introduced a framework that uses face tracking and normalized least mean square (NLMS) adaptive filtering methods to reduce motion artifacts. Lam et al. [21] estimated HR by randomly selecting pairs of traces and performing a majority voting scheme assisted by the skin appearance model, which describes how illuminations and motion artifacts affect the skin’s appearance over time. Although those methods make progress for RGB camera-based HR measurement, the main drawbacks are still yet to be resolved. For example, Li et al. [20] removed the video segments using non-rigid motions, which can lead to inaccurate measurements during HR monitoring due to the absence of partial heartbeat information. Lam et al. [21] repeatedly selected and computed point trace pairs, leading to high algorithm complexity. Besides, we notice that most works focus on adults while infants are much less studied. Moreover, obvious differences exist between the facial features of babies versus those of adults (e.g., babies have much smaller, rounder faces than adults), making it difficult to adapt the existing adult-suitable methods to the infant.

This study proposes a fast HR measurements method focusing on neonates with improved robustness in the presence of motion artifacts. We achieve real-time measurements using an efficient algorithm. To track the neonatal face as the ROI, we focus on the color and the elliptical feature of baby skin. Subsequently, to improve the robustness of HR measurements, we divide the ROI into patches and magnify the subtle color variations for each patch video. Peak detection is then employed for each patch video to obtain candidate HR values. Finally, we apply majority voting to obtain the final HR with the highest probability value. The proposed method improves the ROI information utilization compared with traditional procession (which spatially average whole ROI pixels into one value). Moreover, the majority voting scheme guarantees that patches with weak heartbeat estimations are statistically unlikely to win based on the intuitive assumption that patches with motion artifacts account for a small part of the baby’s face.

The main contributions of this work can be summarized as follows:

1. A novel, fast and robust HR measurement method is proposed for hospitalized newborn infants.
2. The impact of the different ROI patch sizes on the performance is explored, and the optimal ROI patch size that offers satisfactory performance is provided.
3. The performance of the proposed method is validated in comparison with different methods from the same neonatal database.
4. To the best of our knowledge, this is the first work to reduce motion artifact problems for neonatal HR estimation.

## Results

We chose two conditions from the continuous video recordings of nine subjects. One is *rest still* without any kind of motion artifacts, the other is *visible movements* from a camera including head rotation and non-rigid movements. Each state contains 4–6 video segments spanning 1.13–6.56 min. The total duration is 4.21 h, with 2.15 h during *rest still* and 2.06 h during *visible movements*. The detailed information is shown in Table 1.

**Table 1:** Subject information and experimental parameters

Subject Number	Gender	Gestational age (Week + Day)	Age (Day)	Weight (Kg)	Reason for admission	Duration of *rest still* (hour)	Duration of *visible movements* (hour)
Sub1	F	35 + 3	0	2.27	Preterm	0.35	0.45
Sub2	F	40 + 4	5	2.72	Jaundice	0.22	0.23
Sub3	M	38 + 6	16	3.01	Fever	0.20	0.19
Sub4	M	31 + 3	0	1.62	Anhelation	0.22	0.25
Sub5	M	37 + 1	6	2.91	Jaundice	0.26	0.20
Sub6	M	35 + 3	0	2.26	Preterm	0.31	0.25
Sub7	F	36 + 4	1	2.60	Anhelation	0.18	0.17
Sub8	F	39 + 2	13	3.36	Cyanosis	0.16	0.16
Sub9	M	34 + 6	4	2.35	Jaundice	0.23	0.14

### Optimal patch size evaluation

**Fig. 1:**
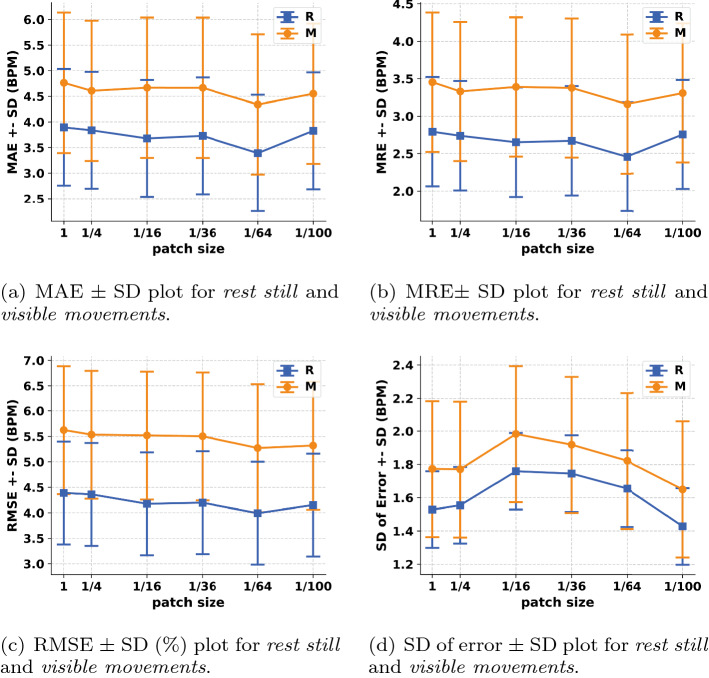
Performances of four metrics—MAE (mean absolute error), MRE (mean relative error), RMSE (root mean squared error) and SD (standard deviation) of error for the proposed method under two different conditions. R represents *rest still*. M represents *visible movements*

To evaluate the performances of different patch sizes during ROI dividing step and choose the optimal patch size that leads to the most accurate measurements, we selected the patch size from 1 to 1/100 of the entire picture and investigated the HR estimation performances of four metrics—mean absolute error (MAE), mean relative error (MRE), root mean squared error (RMSE) and standard deviation (SD) of error. The tendencies are shown in Fig. 1a–d. Overall, we can find that (1) the performance during *rest still* is better than that during *visible movements*; (2) the performances of different patch sizes under different conditions show great similarity. Figure 1a–c present the performances of MAE, MRE, RMSE, respectively, versus patch size under two different conditions. The performance tendencies are relatively similar for different conditions. The three metrics tend to decrease from the patch size $= 1$ to 1/64, indicating the best performance at the patch size of 1/64. When the patch size decreases from 1/64 to 1/100, the three metrics substantially increase. Figure 1d presents the performance of SD (of error) versus patch size under two different conditions. The SD of error tends to increase from a patch size $= 1$ to 1/16, then drops linearly from a patch size = 1/16 to 1/100.

Based on the above results, 1/64 (or 3.84% of the entire ROI) is considered as the optimal patch size leading to the most accurate measurements for the remainder of analysis.

### Individual HR measurements at the optimal patch size

The performances of individual subjects at the optimal patch size (1/64) (leading to the most accurate measurements) during *rest still* (represented as R) and *visible movements* (represented as M) are shown in Table 2. In Table 2, MAE, MRE, RMSE and SD represent mean absolute error, mean relative error, root mean squared error and standard deviation of error, respectively. Our method achieves an average MAE of 3.39 beats per minute (BPM) and MRE of 2.45% during *rest still*. The standard deviation of MAE and MRE during *rest still* are separately 1.14 BPM and 0.73%. Besides, an average MAE of 4.34 BPM and MRE of 3.16% during *visible movements* can be found in the same table, which is slightly higher than that during *rest still*. The standard deviation of MAE and MRE during *visible movements* are 1.37 BPM and 0.93%, respectively. Table 2 displays that our method has a promising performance for most subjects, which verifies the feasibility of the proposed work. However, the performances of subject 2 and 9 are relatively unsatisfying. The possible reason is that some unexpected head translation movements occur during the recording period.

**Fig. 2:**
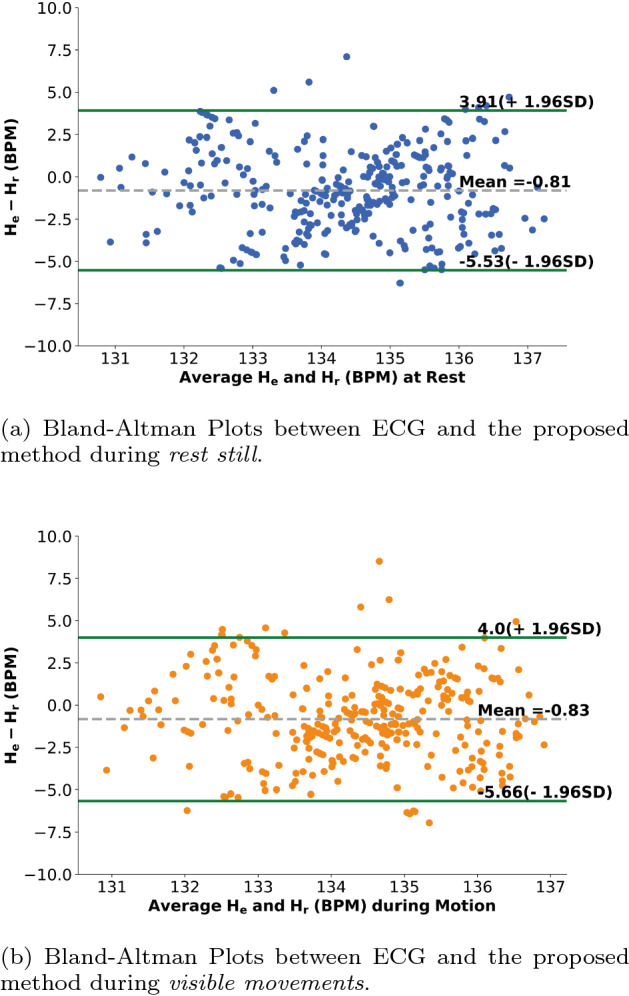
Bland-Altman Plots demonstrating the agreement between 10 s instantaneous HR measurements obtained from one subject under different conditions. The dashed gray line and green lines respectively represent the mean and the 95% limits of agreement. $H_{e}(i)$ represents the estimated HR value for the $i_{th}$ s, $H_{r}(i)$ represents the corresponding HR estimated from the ECG signal

**Fig. 3:**
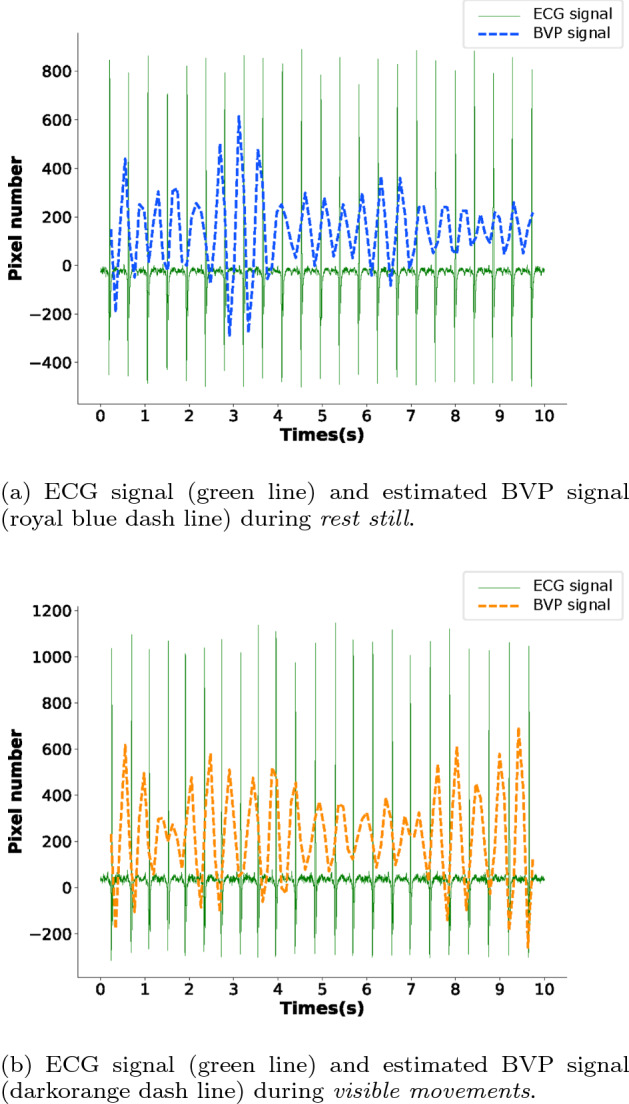
An example of synchronized ECG and estimated BVP signal from 10 seconds of one particular subject under different conditions

The Bland-Altman analysis for the measurements of average HR using an RGB camera under two different conditions is shown in Fig. 2. Bland-Altman plots show that the HR measurements during *rest still* produces a bias of − 0.81 BPM and a standard deviation of the difference that equals 2.41 BPM, indicating that HR is slightly underestimated using our proposed method. Accordingly, the 95% limits of agreements (LoAs) during *rest still* are − 5.53 and 3.91 BPM. For the HR measurements during *visible movements*, the bias is − 0.83 BPM. The standard deviation of the difference is found to be 2.46 BPM, and accordingly, the 95% limits of agreement (LoAs) are − 5.66 and 4.0 BPM. Figure 3 shows examples of recovered blood volume pulse (BVP) signal using synchronized ECG under two different conditions. We can find that: (1) The heartbeat numbers recovered from the BVP and synchronized ECG signals are equal in Fig. 3a, b; (2) The performance during *rest still* is better than that during *visible movements*—the royal blue pulse is more synchronized with the green pulse than that of the darkorange pulse; (3) A clear time shift can be observed from Fig. 3 due to the traveling time of blood from heart to the facial vessels—the blue and darkorange pulses in Fig. 3 (representing the estimated BVP signal from facial vessels) are always later than the green pulses (representing the ECG signal during each heartbeat).

**Table 2:** Metrics performances for individual subjects under two different conditions

	MAE		MRE (%)		RMSE		SD	
	R	M	R	M	R	M	R	M
Sub1	3.88	4.35	2.80	3.21	4.13	5.34	1.69	1.89
Sub2	5.47	7.45	3.82	5.19	5.83	7.76	1.93	2.19
Sub3	3.57	4.24	2.53	3.29	4.25	6.59	1.67	1.49
Sub4	3.04	4.03	2.61	2.75	4.15	3.95	1.57	0.71
Sub5	1.83	3.37	1.38	2.46	2.62	4.94	2.05	1.95
Sub6	2.59	4.35	1.97	3.36	4.04	5.52	1.53	1.89
Sub7	3.48	2.60	2.54	1.92	4.41	3.92	1.34	1.72
Sub8	1.91	3.07	1.39	2.29	2.12	3.69	1.30	1.58
Sub9	4.74	5.60	3.00	3.96	4.35	5.72	1.77	1.34
Average	3.39	4.34	2.45	3.16	3.99	5.27	1.65	1.64
Std. Dev.	1.14	1.37	0.73	0.93	1.01	1.26	0.23	0.41

## Discussion

### Explanations of performance under different patch sizes

The HR estimation results in neonates reveal that, when the ROI patch size equals 1/64, the performance achieves the most accurate level under both conditions (*rest still* and *visible movements*).

The possible reason for this is that: when patch size is greater than 1/64, the divided patch is relatively large, leading to a small group of histogram ranges. For example, when patch size equals 1/4, the whole facial ROI is divided into four patches. In other words, the final instantaneous HR is decided by the histogram containing only four ranges, which is inaccurate given such few ranges considering that the final HR is obtained by averaging the particular range with the highest probability. In contrast, when patch size is less than 1/64, the divided patch is relatively small, which leads to performance degradation in the color magnification step and an unsatisfying HR estimation performance. Specifically, the Eulerian video magnification (EVM) method tracks the pixel variations of a entire picture using Eulerian perspective. When patch size decreases, the Eulerian perspective degenerates to the Lagrangian perspective, which yields the soar of signal noise (as shown in [22]). In particular, when the patch size is the size of one single pixel, the Eulerian perspective completely degenerates to the Lagrangian perspective, and the magnification noise would reach a maximum.

Therefore, patch size selection is crucial for estimating the most accurate HR value—overly large patch sizes lead an increase in deviation during the majority voting step; contrastingly overly small patch size increases the inaccuracy during the color magnification step. To obtain a satisfying performance, researchers should comprehensively consider the influences of video resolution and the ROI proportion of the entire frame. In our opinion, the best patch size is related to the hardware parameters of the RGB camera, such as sampling rate and resolution.

### Comparison with previous methods

We re-implemented five previous methods and tested them on our database under the two different conditions. The performances of different methods (including our previous one) are shown in Table 3. As shown in Table 3, Poh et al. [23] used color-based analysis for non-contact HR measurement. Specifically, they treated the HR signal estimation from the RGB channels as the “cocktail problem” and used independent component analysis (ICA) to separate the underlying HR component from the three obtained channels. Balakrishnan et al. [24] used motion-based analysis to extract HR from videos. They applied principal component analysis (PCA) to estimate the periodic pulse from the head motions of video recordings based on the principle of remote ballistocardiogram (rBCG). Poh et al. [23] and Balakrishnan et al. [24] both used standard face trackers from OpenCV to obtain the ROI, we did not replicate that process in our neonatal database because of the different facial features between adults and infants. Lam et al. [21] introduced the idea of majority voting from facial subregions. They repeatedly selected and computed point trace pairs from the ROI, leading to high algorithm complexity. Chen et al. [13] (our previous work) is the first study to employ EVM in neonatal HR measurements. However, overcoming the neonatal motion artifact problems was not considered. Matthew et al. [25] manually tracked forehead subregion of infants as the ROI using video frames using publicly available software, then applied fast fourier transform (FFT) to the ROI to find the highest power in the spectral domain as the HR.

**Table 3:** Performance comparison among different methods on hospitalized neonatal database under two different conditions

Authors	Methods	MAE		MRE (%)		RMSE		SD		Time (s)	
		R	M	R	M	R	M	R	M	R	M
Poh 2010 [23]	Color-based analysis	6.43	7.32	4.66	5.23	6.86	8.06	1.98	3.51	30.2	30.6
Balakrishnan 2013 [24]	Motion-based analysis	10.09	12.17	7.05	10.21	9.97	12.08	0.86	3.05	30.7	30.1
Lam 2015 [21]	Majority voting	6.57	7.12	4.71	5.49	6.92	8.82	2.08	2.57	840.2	839.8
Chen 2020 [13]	EVM	5.39	6.22	3.78	4.36	5.37	6.92	1.93	2.53	15.1	15.6
Matthew 2021 [25]	Manual ROI + FFT	8.06	9.15	5.59	6.43	8.22	9.82	2.50	1.78	12.1	12.3
Proposed method	EVM + majority voting	3.39	4.34	2.45	3.16	3.99	5.27	1.65	1.84	20.2	20.5

Table 3 confirms that our method is more accurate—the MAE/MRE of the proposed method during *rest still* is 3.39/2.45 BPM, which improves at least 2.00/1.33 BPM compared with the state of the art methods. The MAE/MRE during *visible movements* is 4.34/3.16 BPM, which improves at least 1.88/1.20 BPM compared with previous methods. Moreover, the proposed method is relatively less time-consuming (around 20 seconds to process one minute of video via Python 2019 on an Intel Core i5-9400F@2.90GHz with 16GB of memory), indicating that our method is effective and utilizes low algorithm complexity. Typically, Balakrishnan et al. [24] have the worst performance as the cyclical movement of blood from the heart to head is greatly deteriorated when babies are lying down, which leads to an increase in estimation error.

### Limitations and further improvements

As the first study of improving robustness for HR measurement in a hospital, it still has some perspectives that can be enhanced in the future. First, the proposed method only focuses on head rotation and non-rigid motion problems. When babies have unexpected head translation movements, the ROI dividing step introduces invalid background noise into the ROI, which leads to performance degradation during HR measurement. This issue can be improved in future studies by adaptively switching between multiple cameras or calibrating facial orientations. Second, this paper is based on the assumption that no large objects (which are close to skin color) exist in the video recordings. If these kinds of objects exist in the video recordings, the morphology-based filtering cannot filter it out, which may bring background noise into pure HR signal and increase the HR measurement error. In the future, this issue can be improved by employing advanced techniques for distinguishing between elliptical faces and irregular shapes (such as nipples). Third, this paper only focuses on motion artifact problems in neonatal practical scenarios, other challenges, such as illumination variations, are not considered. Further discussion on HR measurement under different light conditions and the solution of the illumination variation problems are promising subjects of future studies. Finally, this paper only detects HR using an RGB camera, other vital signals (e.g., respiratory rate, heart rate variability, blood pressure and blood oxygen saturation) and multi-modal data (such as video frames from both thermal and RGB cameras), which are also important for health care monitoring, are not considered. In the future, robustly estimating more parameters using different cameras in real-life situations will be taken into account.

## Conclusion

In this work, we present a novel and fast method for neonatal non-contact HR measurement in the presence of motion artifacts in hospitals. This method introduces ROI dividing to improve ROI information utilization and proposes a majority voting scheme to choose the most reliable HR statistically. Since the RGB camera is economical and convenient to operate, the proposed method can expectedly contribute to vital signs (including heart rate, breath rate and blood oxygen saturation) estimation and help reduce neonatal mortality in hospitals.

## Methods

### Subject information and experimental setup

**Fig. 4:**
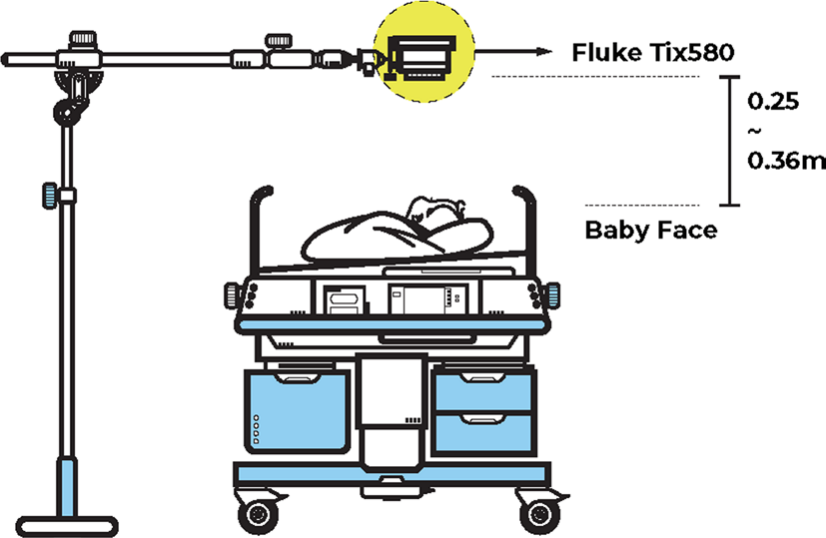
Experimental setup of video recording and corresponding ECG signal acquisition

Nine newborn Chinese babies without known cardiovascular disease or injuries were recruited at the Children’s Hospital of Fudan University. The experiment was approved by the ethics committee of the Children’s Hospital of Fudan University [approval No. (2017) 89]. All subjects’ parents signed a written informed consent. The experimental setup is shown in Fig. 4. This experiment was held in a private room without the interruption of noisy hospital environment. The video recordings were acquired from 9:00 a.m. to 11:30 a.m. for babies to generate a bright and unchanged illumination condition. The subjects were placed 0.25–0.36 m below the camera (TiX580, Fluke Corporation, Shanghai, China) on a comfortable, open bed. The camera recorded two types of color patterns—RGB and thermal. To build a low-cost neonatal HR monitoring system, we only employed an RGB video pattern. The view of RGB videos mainly contain the face of baby and some background surroundings around their face. The RGB videos were recorded at 30 frames per second (fps) with a 640 $\times$ 480 pixel resolution. During the video recordings, a commercially available FDA-approved Nicolet EEG cap with ECG electrode (Phecda, Guangzhou, China) was applied to detect the neonates’ ECG signals at a 500 Hz sampling rate. Before the electrode placement, the skin surface of each baby was softly cleaned with an alcohol pad to improve the signal quality. The video recordings were synchronized with the ECG signals as ground truth to evaluate the performance of the proposed method. When neonates need caretaking activities (e.g., medical examination, cluster feeding, physical examination, etc.), the recorded videos and ECG were suspended simultaneously.

**Fig. 5:**
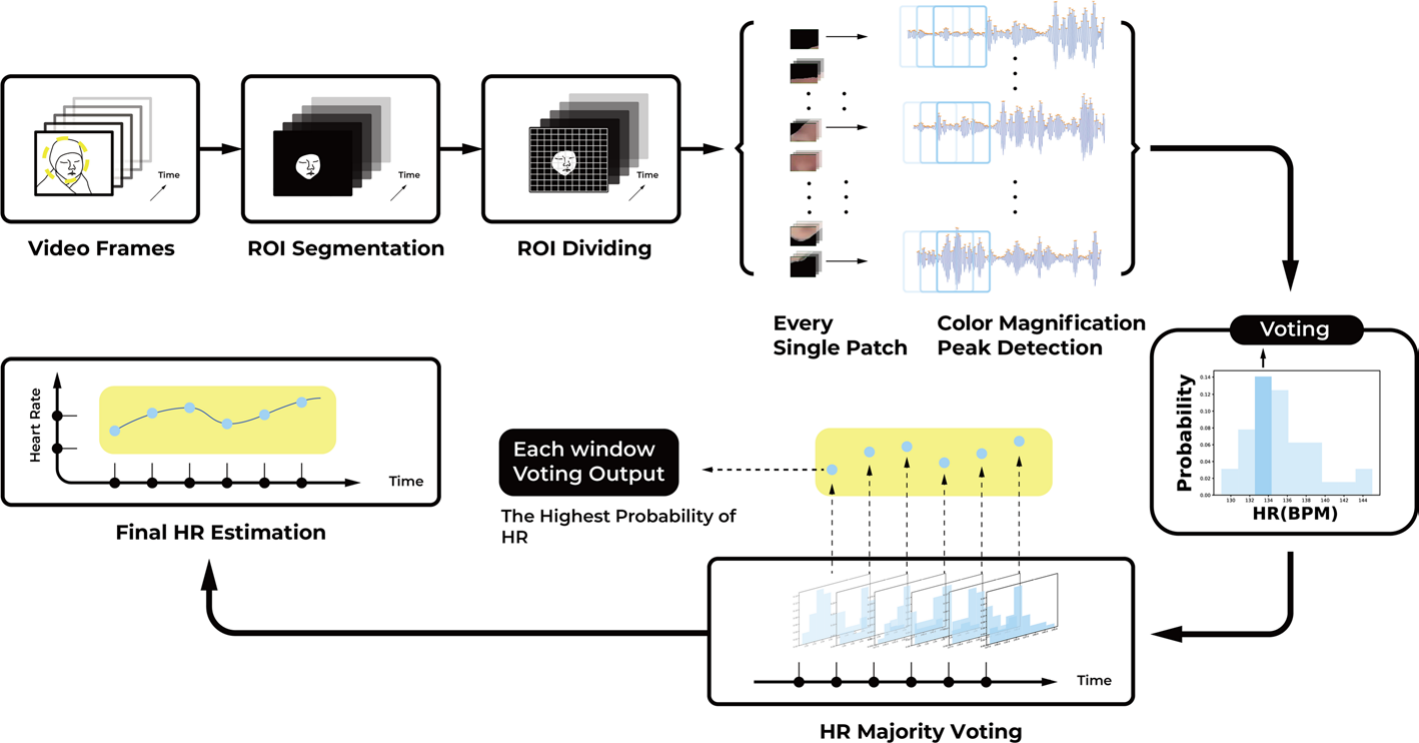
Flowchart of HR measurements steps

Figure 5 presents the main steps of the neonatal HR measurement method. First, the faces of the neonates is extracted as the ROI from video frames using morphology-based filtering. Second, the ROI is divided into non-overlapping patches with specific sizes. For every patch video, the EVM is used to magnify the subtle color changes as BVP signals. Third, the instantaneous HR value of every patch video is calculated via peak detection of the BVP signal and then transformed from peak numbers to BPM. Finally, majority voting is used on the candidate HR pool of all patches to obtain the final HR. The details of the method are explained in the following subsections.

### ROI segmentation

Based on previous studies [26, 27], it is important to select reliable ROI from background noise. There are some classic ROI extraction methods. One is tracking the coordinates of rectangular face locations using face trackers from the Open Computer Vision (OpenCV) library [28] based on Viola and Jones’ [29] algorithm, which is convenient for face detection. However, this off-the-shelf method is limited due to some inherent defects. First, the face tracker can not synchronously track the face when subjects move. Second, the face tracker only finds coarse rectangular facial locations and brings non-face pixels into the ROI. The non-face pixels within rectangle corners inevitably bring background noise. Third, the face tracker, which is suitable for adults cannot fit neonates as the facial features of newborn babies are different from adults (babies’ faces are much smaller and rounder than adult faces. The eyes, nose and mouth between babies and adults are also clearly different).

Another advanced method is locating facial landmarks using the discriminative response map fitting (DRMF) method [30] and then applying kanade-lucas-tomasi (KLT) to track feature landmarks frame by frame. The alternative method solves coarse facial location and motion artifacts while still cannot resolve the neonatal face tracking problem. As such, we adopt a simple but practical method that utilizes the continuity of skin color values in the HSV (hue, saturation and value) color domain. First, we convert video recordings from the RGB color domain to the HSV color space since the skin color in the HSV domain normally ranges in a continuous interval (higher than [0, 10, 60] and lower than [20, 150, 255] in the H, S and V channels for Chinese infants [13, 31]). Then, pixels within that interval are retained, and pixels outside that interval are filtered out. Afterwards, the segmented ROI is transformed back from the HSV color domain to the RGB color domain. Finally, the edges of the ROI are smoothed using morphology-based filtering. Specifically, the open operation (the process of an erosion operation followed by a dilation operation) and close operation (the process of a dilation operation followed by an erosion operation) are utilized in succession to perfect the elliptical boundary of infants’ faces. The advantage of our skin segmenting method is that skin color instead of facial features is primarily considered. Therefore, it is robust no matter what movement the babies make. Another advantage is that it is fast and convenient compared with training a neonatal face classifier [32, 33].

### ROI dividing

After ROI segmentation, the pixel values except for the ROI are set to 0, which means that the background of video recordings is black. The next step is to divide the facial ROI into particular patch sizes. Specifically, we cut the width and height of each frame into 2 (or 4, 6, 8, 10) equal pieces. The size of each single patch is 1/4 (1/16, 1/36, 1/64, 1/100) of the intact frame, which is 25% (11%, 6.25%, 3.84%, 2.78%) of ROI area. We do not need the background patches due to the lack of HR information. Therefore, for a single patch video, if the first frame is totally black (representing an invalid patch without any ROI information), the video will be deleted from the available patch pool. Otherwise, the patch video will be remained. After doing this, patch videos containing heartbeat information are saved for further analysis. Since the motion artifact types in newborns are mainly head rotation and local non-rigid motions (which have no relative head translation), even choosing an ROI area in the first frame retained valid HR information in the following frames.

### Color magnification

To obtain the BVP signals from each patch video, we apply the color magnification method. The principle of color magnification can be explained as follows. We take a one dimension signal undergoing motion as an example, where *I*(*x*, *t*) denotes the intensity of an image at position *x* and time *t*. Since the image undergoes motion, the observed intensities with respect to a displacement function $\delta (t)$ can be expressed as $I \left( x,t \right) = f \left( x + \delta (t)\right)$, where $I\left( x,0 \right) = f\left( x \right)$. The objective of motion magnification is to find the synthesized signal ${\hat{I}}\left( x,t\right) = f\left( x + \left( 1+ \alpha \right) \delta (t)\right)$ for the amplification factor $\alpha$. We apply EVM to amplify the subtle color variations yielded by heartbeat [22]. The EVM method was proposed by Wu et al. in 2012 to reveal temporal variations in videos that is difficult to see with the naked eye, such as the guitar string and the shadow of sun. They propose the Eulerian perspective to track the variations of pixels at a fixed area instead of the traditional Lagrangian perspective which focuses on the movement of specific pixels at each instant. In particular, to intensify the change of signals in a particular space, the Eulerian perspective does not explicitly estimate the movement of individual pixels, but exaggerates the pixel value variation by amplifying temporal color changes at a fixed position. The main steps of the EVM method are described below.

1. Spatial filtering: The first step of the EVM method is decomposing the video frames into different spatial frequency bands and then increasing the temporal signal-to-noise ratio by pooling multiple pixels. To do this, the patch video frames undergo spatial low-pass filtering and downsampling to improve the computational efficiency. The two steps are combined using the full Laplacian pyramid in the EVM method.
2. Temporal filtering: For each spatial band, band pass filtering is performed to extract the variation part of interest. Since infant’s HR range is 110–160 BPM [34], we choose the ideal bandpass filter within 1.8333–2.6667 Hz to directly cut off the frequency band of interest, and avoid amplifying other frequency bands.
3. Amplification: We then choose a magnification factor $\alpha$ of 150 (refer to [13]; the $\alpha$ is normally set at 100–200 for color-based magnification).
4. Signal combination: The magnified signal is added to the original, and the spatial pyramid is collapsed to obtain the final output.

Based on previous studies [16, 23], the green channel has the greatest signal-to-noise ratio and contains the strongest pulsatile signal. Therefore, we spatially average the patch video pixels in the green channel and apply the averaged result for further analysis.

### Peak detection and HR majority voting

After the EVM magnification, the BVP signal is generated by spatially averaging each patch video pixels in the green channel (Fig. 3). To convert the BVP signal of a long period into real-time HR values, we apply peak detection and count the peaks of one minute window, with a sliding window of one second. Thus, the HR sequences of each patch video are obtained for each subject. To reduce the motion artifact problems, we perform a majority voting scheme to choose the final HR sequence from the face patches. In particular, for each specific moment, we choose the average of HR ranges with the highest probability as the final HR from the patch number of HR values. For instance, if one frame is divided into 64 patches, we draw a histogram with 64 HR values and choose the mean of HR ranges corresponding to the highest peak of the histogram. Since the motions only account for small parts of the baby’s face, (which is unlikely to win during majority voting), this scheme improves the robustness of neonatal HR measurements against motion artifacts.

### Validation methodology

We estimate HR from video frames and calculate synchronized ECG during peak detection using a one minute window and a one second sliding window. The estimated HR value for the *i*th second is denoted as $H_{e}(i)$. The corresponding HR estimated from the ECG signal is denoted as $H_{r}(i)$. To conduct a fair comparison between our method for neonatal HR measurement and previous ones applied for adult HR measurement, the MAE, MRE, RMSE and SD of error are used to evaluate the performance of non-contact HR measurements. Details on the four metrics definition are shown in Table 4.

**Table 4:** Metrics for HR measurements

Metric	Definition	Detail description
MAE	Mean bbsolute Error	$\frac{1}{N}\sum \limits _{i = 1}^N {|{H_{e}}(i) - {H_{r}}(i)|}$
MRE	Mean relative Error	$\frac{1}{N}\sum \limits _{i = 1}^N {|\frac{{{H_{e}}(i) - {H_{r}}(i)}}{{{H_{r}}(i)}}|} \times 100\%$
RMSE	Root mean squared error	$\sqrt{\sum \limits _{i = 1}^N {\frac{{{{({H_{e}}(i) - {H_{r}}(i))}^2}}}{{N}}} }$
SD	Standard Deviation	$\sqrt{\frac{1}{N}\sum \limits _{i = 1}^N {({H_{e}}(i) - {H_{r}}(i) - \mu )}^{2} }$
		$$\mu =\frac{1}{N}\sum \limits _{i = 1}^N ({H_{e}}(i) - {H_{r}}(i))$$

## Data Availability

The datasets used and/or analyzed during the current study are available from the corresponding author on reasonable request.

## Abbreviations

HR: Heart rate
ROI: Region-of-interest
ECG: Electrocardiogram
BPM: Beats per minute
rPPG: Remote photoplethysmography
PPG: Photoplethysmography
NLMS: Normalized least mean square
MAE: Mean absolute error
MRE: Mean relative error
RMSE: Root mean squared error
SD: Standard deviation
LoAs: Limits of agreement
BVP: Blood volume pulse
EVM: Eulerian video magnification
ICA: Independent component analysis
PCA: Principal component analysis
rBCG: Remote ballistocardiogram
FFT: Fast fourier transform
fps: Frames per second
OpenCV: Open Computer Vision
DRMF: Discriminative response map fitting
KLT: Kanade–lucas–tomasi
